# The effect of metal substitution in CsSnI_3_ perovskites with enhanced optoelectronic and photovoltaic properties

**DOI:** 10.1039/d1ra07609d

**Published:** 2021-12-13

**Authors:** M. N. Islam, J. Podder, M. L. Ali

**Affiliations:** Department of Physics, Bangladesh University of Engineering and Technology Dhaka-1000 Bangladesh jpodder59@gmail.com; Department of Physics, Pabna University of Science and Technology Pabna-6600 Bangladesh

## Abstract

Non-toxic lead-free halide metal perovskites have gained significant interest in photovoltaic and optoelectronic device applications. In this manuscript, we have studied the structural, electronic, mechanical, and optical properties of eco-friendly cubic CsSn_1−*x*_Cu_*x*_I_3_, (*x* = 0, 0.125, 0.25, 0.5, 1) perovskites applying first-principles pseudopotential-based density functional theory (DFT). Cu-doped CsSnI_3_ has a large impact on the band gap energy *viz.* the transition of direct band gap towards the indirect band gap. The mechanical properties demonstrate that the pristine and Cu-doped CsSnI_3_ samples are mechanically stable and their ductility is enhanced by Cu doping. The mechanical stability and ductility favors the suitability of pure and Cu-doped samples in the thin film industry. The absorption edge of Cu-doped CsSnI_3_ moves towards the lower energy region in comparison with their pure form. In addition, the high dielectric constant, high optical absorption, and high optical conductivity of Cu-doped CsSnI_3_ materials suggests that the studied materials have a broad range of applications in optoelectronic devices, especially solar cells. A combined analysis of the structural, electronic, mechanical and optical properties suggests that CsSn_1−*x*_Cu_*x*_I_3_, (*x* = 0, 0.125, 0.25, 0.5, 1) samples are a suitable candidate for photovoltaic as well as optoelectronic device applications.

## Abstract 2

Non-toxic lead-free halide metal perovskites have gained significant interest in photovoltaic and optoelectronic device applications.

## Graphical abstract

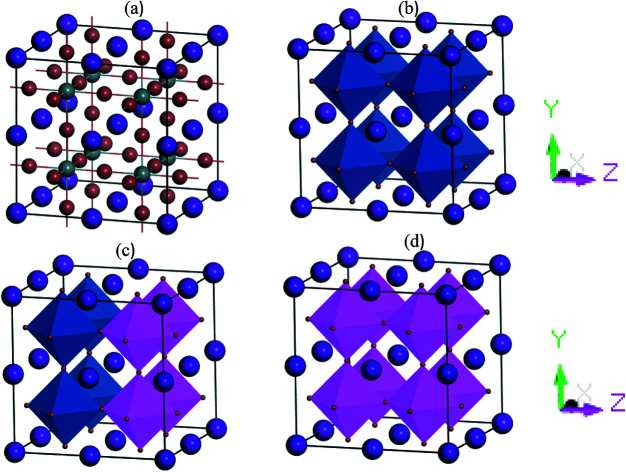

## 1. Introduction

In the last decades, lead-free metal halide perovskites have been used in versatile applications like photovoltaics, light-emitting diodes, lasers, and optoelectronics because of their outstanding electronic and optical properties.^1–5^ Practical applications of metal halide, CsSnI_3_ perovskites have increased to a large scale owing to their unique optoelectronic properties including large tunable direct band gap with high light absorption potential, outstanding charge carrier mobility, low recommendation rate, strong optical absorption, and high dielectric constant.^6–11^ The well-known chemical formula of a metal halide is ABX_3_, where A refers to a cation, B represents a divalent material and X stands for a halogen anion.^12,13^ The cubic CsSnI_3_ perovskite is composed of corner-sharing SnI_6_ octahedral which forms a three dimensional network, where the A-site cations reside in the 12-fold coordinated voids to preserve charge neutrality. In the recent years, the efficiency of perovskite solar cells has increased considerably from 3.8% to over 22% due to intense efforts on the optimization of perovskite layers. CsPbX_3_ (X = halide ions) are outgoing as a member of promising light emitters due to small size, tunable band gaps from the violet to near-infrared and immensely narrow full width at half-maximum.^14–18^ A large number of researchers are avoiding lead halide perovskite material due to toxicity and searching new metal halide perovskites for applications in optoelectronic and photovoltaic. Roknuzzaman *et al.* reported that metal cubic structure perovskites CsBX_3_ (B = Sn, Ge, and X = Br, Cl, I) samples have better applications for an optoelectronic especially solar cell in compared to lead halide perovskites CsPbX_3_ (X = Br, Cl, I).^19^

In recent years, metal halide perovskites fulfill optoelectronic demands in the commercial market. Due to mixing halide ions with perovskites materials, improve material stability, tunable band gap and enhanced photoluminescence properties. Metals doped halide perovskites like MAPbI_3−*x*_ Br_*x*_, MAPbBr_3−*x*_ Cl_*x*_, MA_0.15_FA_0.85_ Pb (I_0.85_Br_0.15_) and Cs_0.17_FA_0.83_Pb (I_0.6_ Br_0.4_) have been greatly improved structural, electrical, optical properties.^20–22^

Metals substitution doping with single halide CsBX_3_ (B = Sn, Ge, and X = Br, Cl, I) perovskites are used in solar cell devices due to faster electrons transport occurred within cation and divalent materials. In spite of breakthroughs, CsSnI_3_ metal halides have no use in device applications, because of their poor stability. For increasing device efficiency, researchers are trying to improve the instability of the perovskite light absorber.^23,24^ Lead-free cesium tin halide, (CsSnI_3_) is highly desirable for device application especially solar cells. CsSnX_3,_ (X = Br, Cl, I) perovskites are promising candidates, especially CsSnI_3_ due to the semiconductor properties.^25,26^ Lead free-metal halide CsSnI_3_ has direct band gap energy of 0.44 eV, while the Cu-doped CsSnI_3_ samples have a large impact on band gap energy due to reducing the electronic band gap energy. The maximum valence band and the minimum conduction bands are staying at the same *k*-points in the Brillouin zone, which is indicating the pure CsSnI_3_ sample has direct band gap nature.^8,24^ The CsSn_1−*x*_Cu_*x*_I_3_, (*x* = 0.125, 0.25, 0.5, 1) samples have indirect band gap energy of 0.59 eV for CsSn_0.875_Cu_0.125_I_3_, 0.48 eV for CsSn_0.75_Cu_0.25_I_3_, 0.42 eV for CsSn_0.5_ Cu_0.5_ I_3_ and 0.29 eV for CsCuI_3_. Notable, 100% Cu-doped CsSnI_3_ sample has indirect band gap energy, with 0.29 eV, reduced the band gap approximately 48.2% from pristine CsSnI_3_ sample. In case of Cu-doped CsSnI_3_ sample, the band gap energy is transferred towards direct to indirect due to intra-band and inter-band transition that occurred in the CsSnI_3_ lattice network. Raman and Hossain attempted to substitute metals at the G-site of CsGeCl_3_ halide to simply improve the absorption over the range of solar energy.^25^ Transition metal Ni-doping in CsGeCl_3_ increases its band gap energy and shrinkage the optical absorption due to the Moss–Burstein effect.^27–29^ In this manuscript, we are addressing the effects of Cu-doped CsSnI_3_ for optoelectronic and photovoltaic applications. We have applied density functional theory (DFT) to calculate electronic, mechanical, and optical properties. A combined analyzed manifested that Cu-doped CsSnI_3_ is a potential candidate material for applications in photovoltaic and optoelectronic devices, especially solar cells.

## 2. Theoretical methodology

The theoretical simulations of lead-free metal halide CsSn_1−*x*_Cu_*x*_I_3_, (*x* = 0, 0.125, 0.25, 0.5, 1) samples were studied using pseudo-potential density functional theory (DFT) simulations of the supercell approach. In this manuscript, 2 × 2 × 2 supercell model is constructed for all simulations. The supercell of CsSnI_3_ contains 40 atoms including eight Cs atoms, eight Sn atoms, and 24 I atoms. All of the calculations in this study were performed by material studio 8.0 based on density functional theory.^27,30^ For geometry optimizations, we employed general gradient approximation (GGA) exchange-correlation function, while the Perdew–Burke–Ernzerhof (PBE)^31^ was selected to conduct the simulation to gain the electronic behaviors in the sample and exact formation energy. The cutoff energy of the plane wave basis set was used at 700 eV for pristine and Cu-doped CsSnI_3_ samples. We have employed 10 × 10 × 10 gamma centered *k*-points for pure and Cu-doped CsSnI_3_ samples. A scissor value (0.68 eV) was applied for the calculations of absorption, conductivity, and dielectric function. A scissor value (0.68 eV), a disparity, between theoretical (0.59 eV) and experimental (1.27 eV) band gap of CsSnI_3_. The crystal structure is completely optimized through a change in *k*-points and cut-off energy and finally, we have found ground state energy in the studied samples. The lattice parameters and coordinates are varying in time under the Broyden–Fletcher–Goldfarb–Shanno (BFGS) algorithm method.^32^ The unit cell parameters and atomic relaxations were accomplished by the residual forces under 0.03 eV Å^−1^. With the CASTEP code, the mechanical properties are calculated by using the finite-strain theory.^33,34^ Stress tensor has six stress components *σ*_ij_ for each strain *δ*_j_ applied to the unit cell.

## 3. Results and discussion

### 3.1. Structural properties and phase stability

**Fig. 1:**
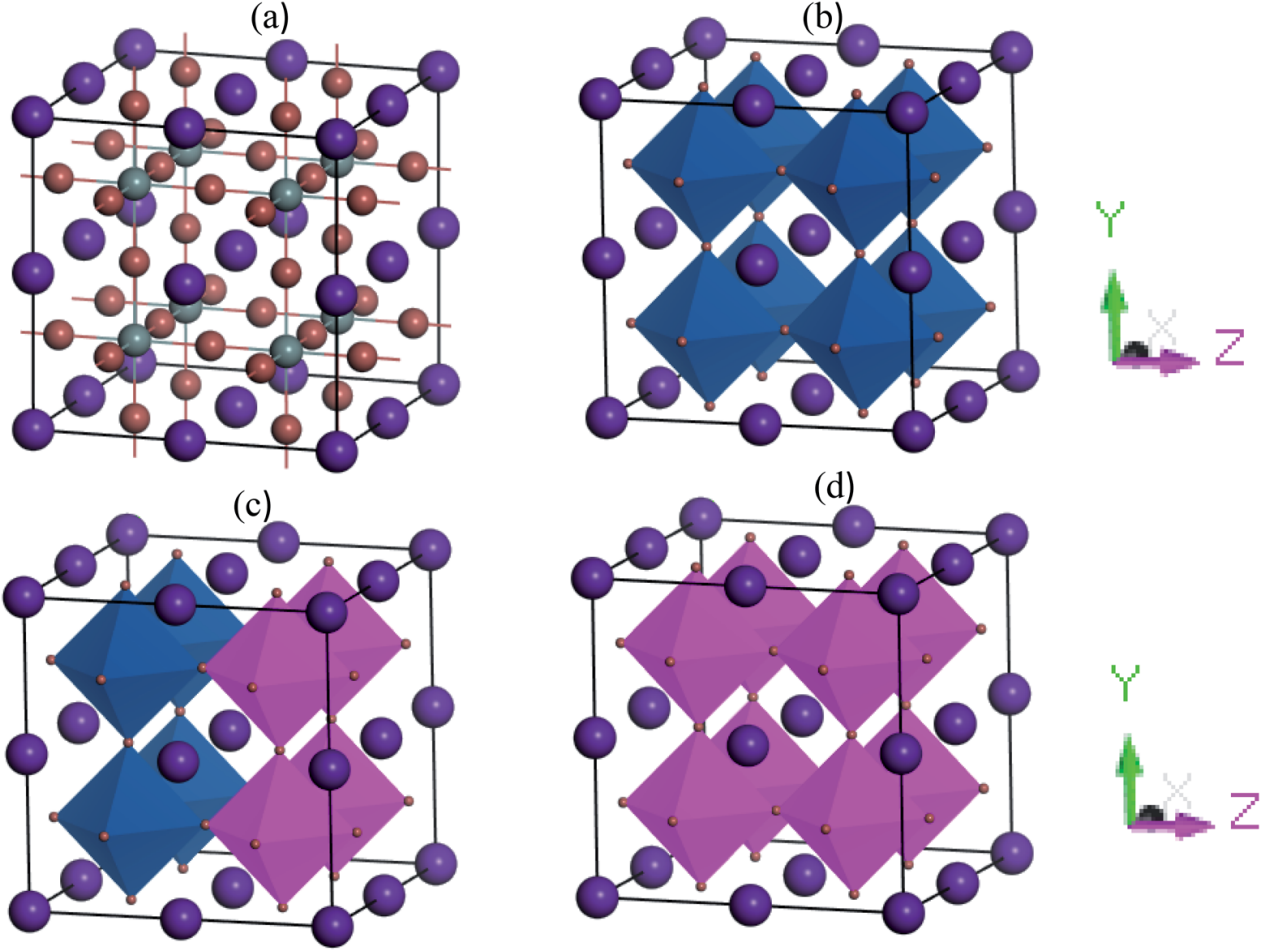
2 × 2 × 2 supercell structures of (a) CsSnI_3_ (balk-and-stick) (b) CsSnI_3_ (polyhedral), (c) CsSn_0.5_Cu_0.5_I_3,_ and (d) CsCuI_3_.

The cubic metal halide CsSnI_3_ perovskites have space group *Pm*3̄*m* (no. 221). In the unit cell, Cs atoms are located on the face-centered position (0 0 0) fractional coordinates, the Sn atoms occupy the body-centered position with fractional coordinates (0.5 0.5 0.5) and I atom located on the face-centered positions with fractional coordinates (0 0.5 0.5). In supercell structures, the simulated equilibrium lattice parameter *a*, and unit cell volume, *V*, are well-matched with experimental as well as theoretical published results. Fig. 1(a–d) shows the cubic structures 2 × 2 × 2 supercell of pure and Cu-doped CsSnI_3_. The cell volume and lattice parameters are decreased with Cu doping concentrations due to lattice strain occurred in random directions.

Phase stability is more essential for materials. To be stable, the materials have to fulfill some special criteria. Firstly, for mechanical stability, a material must have full-filled elastic moduli conditions. The second one is phase stability. In a single halide perovskites material, phase stability is calculated by the tolerance equation.^35^1
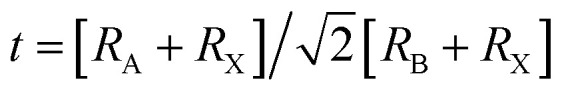
where, *R*_A_, *R*_B,_ and *R*_X_ are represented for the ionic radius of A, B, and I atoms. For stability, the tolerance factor range must be lying in between 0.813 to 1.107. Table 1. Shows that pure and Cu-doped CsSnI_3_ samples full fill phase stability conditions. In the case of 100% Cu-doped CsSnI_3_ sample, the stability is increased compared to pure CsSnI_3_ sample.

Xiao Feng *et al.* reported that the CsSnI_3_ sample has poor stability and can't be suitable for devices.^24^ Notably, the Cu-doped CsSnI_3_ samples have increased phase stability. To gain phase stability, we have employed the Shannon ionic radius. Finally, we concluded that the Cu-doped CsSnI_3_ samples may have potential applications for device purpose.

The formation enthalpy is calculated by the following equations^36,37^

For an un-doped system,2
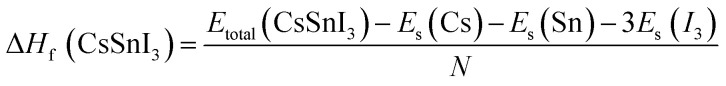
For doped system3
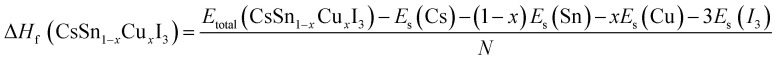
Herein, Cu doping concentrations are varying as *x* = 0.125, 0.25, 0.5, 1. In eqn (2) and (3), *E*_s_(Cs), *E*_s_(Sn), *E*_s_(Cu), and *E*_s_(I) are the energy of Cs, Sn, Cu, and I atoms, respectively, whereas *E*_tot_(CsSn_1−x_Cu_*x*_I_3_) represents the unit cell, total energy, and *N* is the number of atoms in the unit cell. Herein, we have calculated formation energy to see thermodynamic stable nature's of pure and Cu-doped CsSnI_3_ samples. The formation enthalpy values are presented in Table 1. Moreover, the calculated formation enthalpy (Table 1) shows negative values for both pure and Cu-doped CsSnI_3_ halides, which is confirming their thermodynamic stability.

**Table 1:** Optimized structural parameters, formation enthalpy and phase stability for CsSn_1−*x*_Cu_*x*_I_3_ (*x* = 0, 0.125, 0.25, 0.5, 1) halide perovskites compared with experimental and previous theoretical results

Samples	*a*_0_ (Å)		*V*_0_ (Å^3^)		Δ*H*_f_	*t*
	This study	Ref.	This study	Ref.	This study	This study
CsSnI_3_	6.27	6.24^a^, 6.2^c^	247.44	238.9^b^	−2.03	0.99
CsSn_0.875_Cu_0.125_I_3_	6.24	—	243.37	—	−7.24	—
CsSn_0.25_Cu_0.75_I_3_	6.20	—	238.80	—	−7.28	—
CsSn_0.5_Cu_0.5_I_3_	5.98	—	213.91	—	−1.00	—
CsCuI_3_	5.68	—	183.97	—	−2.52	1.02

a Ref. 38.

b Ref. 8.

c Ref. 39.

### 3.2. Electronic properties

**Fig. 2:**
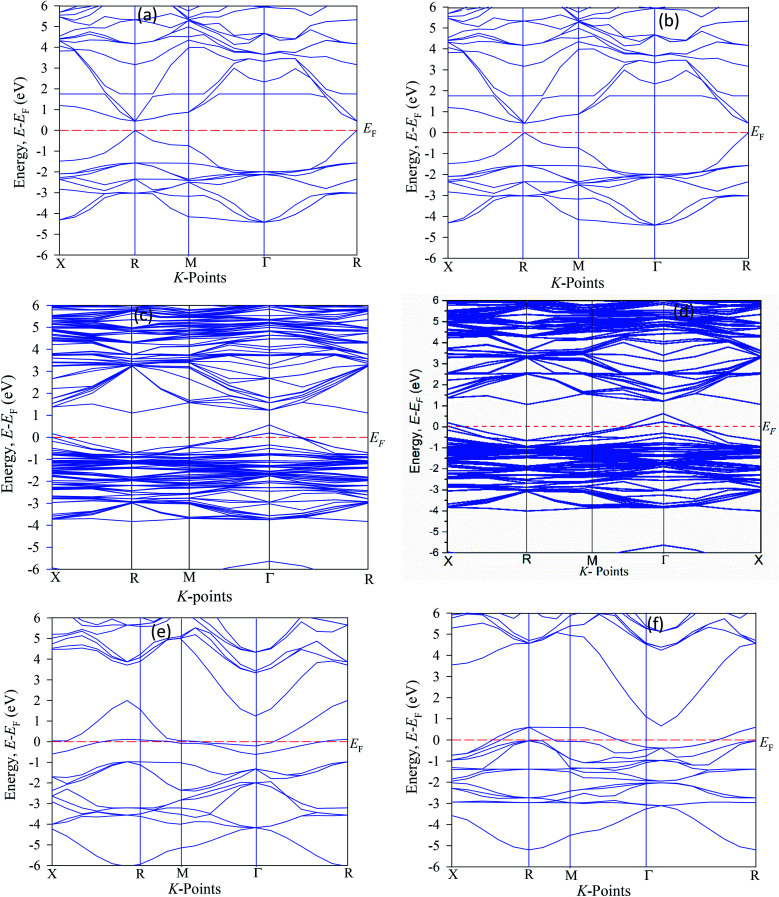
The band structure diagram of CsSnI_3_ (a) pure unit cell, (b) pure supercell, (c) CsSn_0.875_ Cu_0.125_I_3_ (d) CsSn_0.75_ Cu_0.25_I_3,_ (e) CsSn_0.5_ Cu_0.5_I_3,_ and (f) Cs CuI_3._

To see the electronic behaviors of pure and Cu-doped CsSnI_3_ perovskite, we have simulated the electronic band energy along with high symmetry points. The calculated band energy structures are shown in Fig. 2(a–f). The simulated band structures of the pure CsSnI_3_ have direct band gap of 0.44 eV, where the maximum valence band and minimum conduction bands are staying at similar *k*-points. The single cell band gap has small difference from the supercell structure (eight times) band gap. Note that band gap values for unit cell are 0.442 eV and 2 × 2 × 2 supercells of CsSnI_3_ for 0.445, which suggesting the good convergence of the orbital fluctuations.^20^ It is seen that the simulated band gap value underestimates the experimentally calculated band gap values of 1.27 eV.^40^ Several researchers reported that the hybrid potential HSC (Heyd–Scuseria–Ernzerhof) methods are perfect for exact band gap measurements, although this potential is not fit for estimate samples.^13,19^ The experimental calculated band gap value differs from the theoretical band gap due to the limitations of GGA method. However, our work focuses only on the reduction of electrical band gap of Cu-doped CsSnI_3_ and ignores the band gap error for the GGA method. The present band gap energy values are good in agreement with other publication.^40^ It is noteworthy that Cu-doped CsSnI_3_ samples appear in intermediate states.

**Fig. 3:**
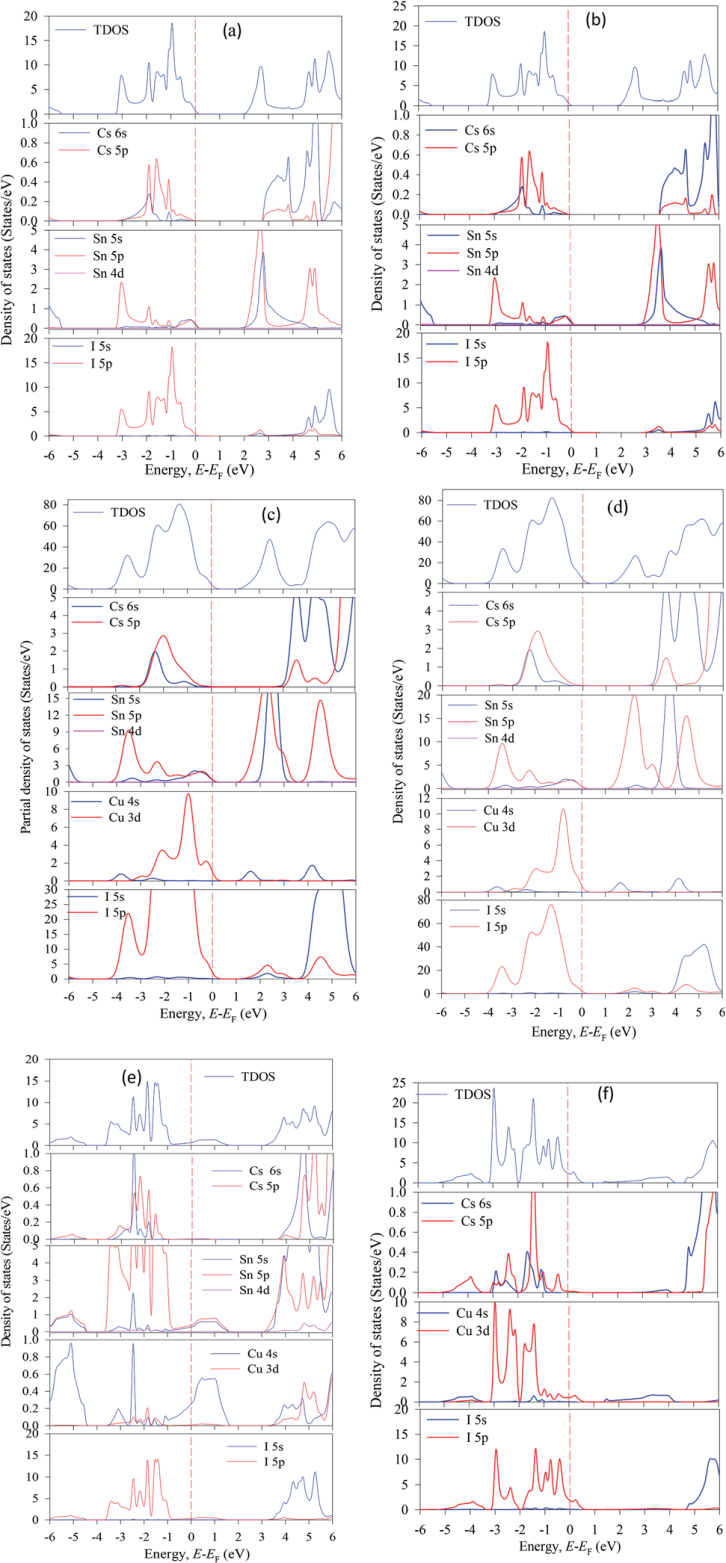
The total and partial density of states of CsSnI_3_, (a) pure unit cell, (b) pure supercell, (c) CsSn_0.875_ Cu_0.125_I_3_, (d) CsSn_0.75_ Cu_0.25_I_3_, (e) CsSn_0.5_ Cu_0.5_I_3_ and (f) Cs CuI_3_.

The valence energy states are expanded into the higher energy region due to the valence band into the Fermi level can cross the transition of electrons from the valence band to the conduction band. Here, shifting of the Fermi level into the valence band can be described as the negative Burstein shift. However, band gap energy is calculated by the maximum valence band gap to the minimum conduction band gaps. It can be seen from Cu-doped CsSnI_3_ samples, the valence band (VB) maximum and conduction band (CB) minimum are lying at dissimilar *k*-points which is indicating the samples have indirect band gap natures. The indirect band gap nature samples have strong absorption and long charge carrier lifetime rather than direct semiconductor samples. The indirect band figure indicates that the electron cannot move to the highest-energy states in the valence band to the conduction band, without in change *k*-points momentum energy. Generally, the indirect nature semiconductors are a promising candidate for photovoltaic device applications. The calculated band gap values are tabulated in Table 2 along with previously published theoretical and experimental results. The calculated band structure suggests that the pattern of the band gap is affected by the Cu-doping concentrations. The total density of states (TDOS) and partial density of states (PDOS) of pure and Cu-doped CsSnI_3_ samples are presented in Fig. 3(a–f). As shown in the partial density of states figure the valence band is mostly composed of Cu-3d and I-6s orbital with a small contribution of Cs-6s and Cs-3p states. The high energy band is mainly dominated by Cu-3d orbital with a small contribution of Cs-6s and Cs-5p electrons. The band structure identifies that the difference between valences band maximum to the conduction band minimum in a sample. The TDOS shape of Cu-doped CsSnI_3_ becomes broader than that of pure CsSnI_3_, which indicates that the electronic non-locality is more because of the reduction of crystal symmetry.^41^ The conduction band energy is mostly attributed to the Cu atom due to CB shifts towards the lowest energy states. A flat peak is seen in the conduction band because Cu-3d states are generated by new dopants energy states. It is observed from Cu-doped CsSnI_3_ samples, the impurity energy states appear in the partial density of states. This intermediate state appears electronic band structure, which is essential for electrons transition between conduction bands to valence bands.

**Table 2:** The energy band gaps (*E*_g_) of CsSn_1−*x*_Cu_*x*_I_3_ (*x* = 0, 0.125, 0.25, 0.5, 1)

Samples	Types of band gap	Present work	Band gap value, *E*_g_ (eV)	
			Exp. ref.	Theo. ref.
CsSnI_3_ (unit cell)	Direct	0.442	1.27^a^	0.30^b^, 0.59^b^, 0.38^c^
CsSnI_3_ (supercell)	Direct	0.445	—	—
CsSn_0.875_Cu_0.125_I_3_	Indirect	0.59	—	—
CsSn_0.25_Cu_0.75_I_3_	Indirect	0.48	—	—
CsSn_0.5_Cu_0.5_I_3_	Indirect	0.42	—	—
CsSnCuI_3_	Indirect	0.29	—	—

a Ref. 34.

b Ref. 28.

c Ref. 42.

#### 3.2.1 Mulliken population analysis and charge density distribution

The effective atomic charge, bond population and bond length in a crystalline solid can be obtained from Mulliken population analysis which gives insight into the charge distribution of electron in various parts of bond, level of covalence and bond length.^43,44^ Mulliken effective charge can be analyzed by the following equation^44^4
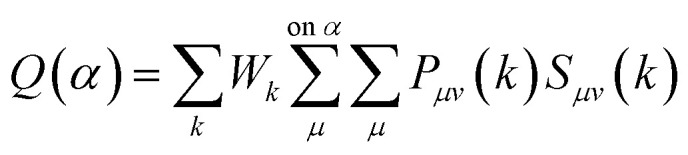
Here, *P*_*μv*_ represents an element of the density matrix and *S*_*μv*_(*k*) is the overlap matrix.

The overlap population between two atoms *α* and *β* can be expressed using the following equation^44^5
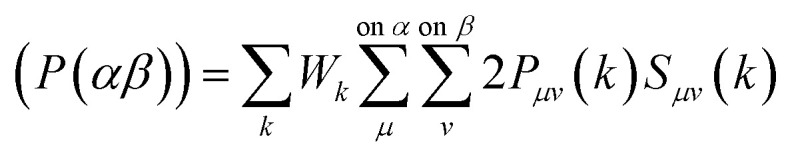


Noticeably, the Mulliken effective charges of the individual Cs, Sn, I, and Cu atoms are found to be reasonably smaller than their formal ionic charges, which are +1, +4, −1, and +2, respectively.

To see the difference between Mulliken effective charge and formal ionic radius, we have applied the Shannon ionic radius. The difference between Mulliken effective charges and formal ionic radius indicates that the CsSn_1−*x*_Cu_*x*_I_3_ (*x* = 0, 0.125, 0.25, 0.5, 1) samples have mixed ionic and covalent bonds (Table 3).

**Table 3:** Mulliken effective charges of individual atoms, bond population and bond lengths of pure and CsSn_1−*x*_Cu_*x*_I_3_ (*x* = 0, 0.125, 0.25, 0.5, 1)

Samples	Species	Effective valence charge (e)	Bonds	Bond population	Bond length (Å)
CsSnI_3_	Cs	0.53	Sn–I	0.07	3.139
	Sn	0.32			
	I	−0.28			
CsSn_0.875_Cu_0.125_I_3_	Cs	0.52	Cu–I	0.08	3.02
	Sn	0.32	Sn–I	0.02	3.11
	I	−0.26	Cs–I	0.33	7.64
	Cu	−0.36			
CsSn_0.75_Cu_0.25_I_3_	Cs	0.51	Cu–I	0.06	2.96
	Sn	0.31	Sn–I	0.01	3.09
	I	−0.26	Cu–Cs	0.04	7.59
	Cu	−0.42			
CsSn_0.5_Cu_0.5_I_3_	Cs	−0.13	Cu–I	0.25	2.87
	Sn	0.30	Sn–I	0.08	3.199
	I	0.21	Cu–Cs	0.28	3.199
	Cu	−0.49			
CsCuI_3_	Cs	0.32	Cu–I	0.23	2.843
	Cu	−0.65	Cu–Cs	0.64	4.92
	I	0.11			

The effective valence charge is reduced with Cu-doping concentrations. The level of covalence Cu-doped CsSnI_3_ reduced due to the effect of on-site Coulomb interaction. The positive value of bond population refers to the high degree of covalence, whereas the small bond population identifies a high degree of ionicity in the covalence bond.^45^ Moreover, the simulated bond populations of Cu–I are found to be higher than the Sn–I. The bond length is decreased in pristine CsSnI_3_ compared to Cu-doped CsSnI_3_ due to ionic radius mismatch between Sn and Cu atoms.

**Fig. 4:**
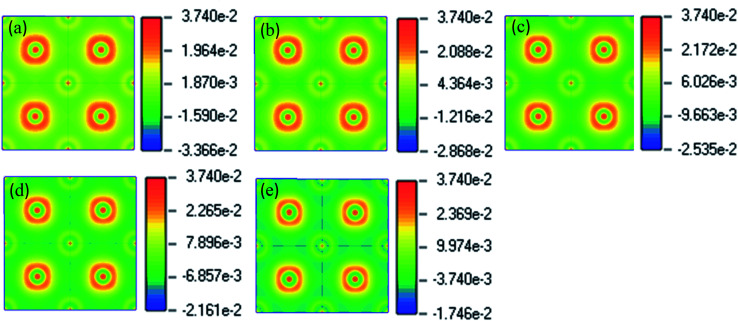
The electric charge density (a) CsSnI_3_, (b) CsSn_0.87.5_Cu_0.125_I_3_, (c) CsSn_0.75_Cu_0.25_I_3,_ (d) CsSn_0.5_Cu_0.5_I_3_ and (e) CsCuI_3_.

To see the charge distribution and bonding nature of pristine and Cu-doped CsSnI_3_, we analyzed charge density distribution and presented it in Fig. 4(a–e). Spherical shape charge distribution exists in the pure and Cu-doped CsSnI_3_. Sn and Cu atoms are bonded covalently with the I atom. The electron clouds around Sn, Cu, and I atoms are distorted towards, which indicating the covalent bond natures. Fig. 4 shows the almost similar charge distribution and bonding character of pure and Cu-doped CsSnI_3_ perovskites.

**Fig. 5:**
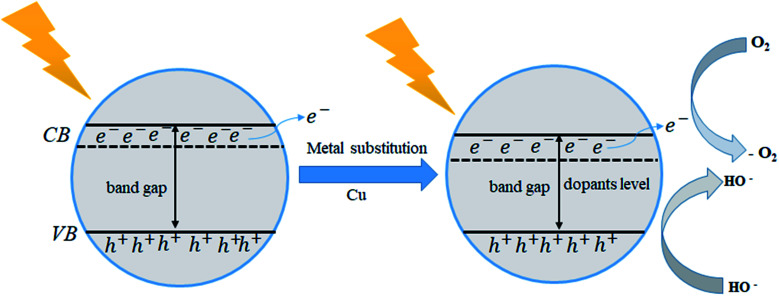
Schematic plot of band energy alignment of the photo catalytic of CsSnI_3_.

Photo-catalytic is an essential parameter to identify the device efficiency of optoelectronic and photovoltaic applications. Tin-based halides CsSnI_3_ have more response to photo-catalytic materials. Cu-doped CsSnI_3_ samples, photo-catalytic activity tends to increase in comparation to pristine CsSnI_3_. Charge carrier mobility transition samples have more photocatalytic activities efficiency. In this paper, we found that the CsSnI_3_ has a direct electrical band gap of 0.44 eV and Cu-doped CsSnI_3_ the sample is transferred from direct band gap energy into indirect. The indirect electrical band gap of CsSnI_3_ samples shows a long lifetime of photo-excited electrons and holes comparsion than direct electrical band gap semiconductors due to the direct apartness of photogenerated electrons from the CB to the VB of a semiconductor is not possible. The excited electrons from the valence band (VB) are injected into the conduction band (CB), which changes the gap energy in Cu-doped CsSnI_3_ samples. The band energy alignment of photocatalytic is shown in Fig. 5. It gives the influence of the separation of photo-generated electron–hole pairs, as well as favors in migration of photo-excited carriers and processing photo-catalysis. Cu-doped CsSnI_3_ samples introduction of new dopant energy levels that effectively changes the band gap energy of the photo-catalyst. This work would be suitable for optoelectronic and photovoltaic device applications.

### 3.3. Mechanical properties

**Fig. 6:**
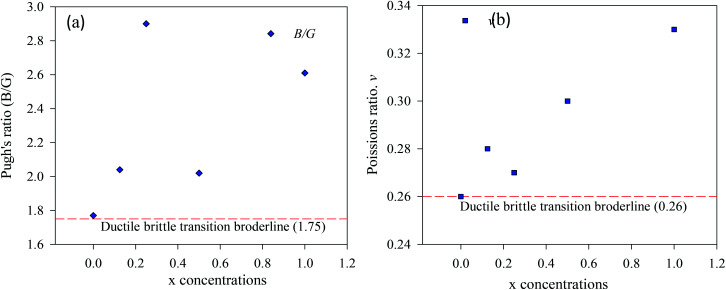
The ductile and brittle behavior (a) Pugh's ratio, (b) Poisons ratio of CsSn_1−*x*_Cu_*x*_I_3_, (*x* = 0, 0.125, 0.25, 0.5, 1).

The three independent elastic moduli for pure and Cu-doped CsSnI_3_ perovskites are simulated by the finite strain theory.^43,46^ The single and polycrystalline elastic properties are simulated *via* CASTEP code material studio 8.0 and tabularized in Tables 4 and 5. The simulated polycrystalline properties for CsSnI_3_ are good to coincide with the previously published paper.^34^ For cubic symmetry criteria, the simulated elastic modulus of pure and Cu-doped CsSnI_3_ compound should satisfy the following conditions:^44,47^*C*_11_ + 2*C*_12_ > 0, *C*_44_ > 0 and *C*_11_ − *C*_44_ > 0. The simulated elastic modulus for pure and Cu-doped CsSnI_3_ full filed the mechanical stability criteria, which indicating that pristine and Cu-doped CsSnI_3_ samples are mechanically stable. The quantity *C*_12_–*C*_44_, is defined as Cauchy pressure,^45,48^ which identifies the brittle/ductile nature of a sample. The simulated Cauchy pressure is positive values, which ensures that the pristine and Cu-doped CsSnI_3_ samples are ductile natures (Fig. 6).

**Table 4:** Calculated elastic constants *C*_*ij*_ (in GPa) of CsSn_1−*x*_Cu_*x*_I_3_ (*x* = 0, 0.125, 0.25, 0.5, 1)

Samples	*C*_11_	*C*_12_	*C*_44_	*C*_12_–*C*_44_	Reference
CsSnI_3_	33.59	16.79	8.65	8.14	—
CsSn_0.875_Cu_0.125_I_3_	22.74	6.68	4.47	2.22	—
CsSn_0.25_Cu_0.75_I_3_	19.02	5.89	3.97	1.92	—
CsSn_0.5_Cu_0.5_I_3_	28.23	12.24	8.88	4.00	—
CsCuI_3_	33.59	16.79	8.68	8.11	—

**Table 5:** The evaluated mechanical parameters of CsSn_1−*x*_Cu_*x*_I_3_ (*x* = 0, 0.125, 0.25, 0.5, 1)

Samples	*B* (GPa)	*G* (GPa)	*Y* (GPa)	*B*/*G*	*v*	Reference
	16.56	9.03	22.92	1.83	0.26	34
CsSnI_3_	15.57	8.78	22.19	1.77	0.26	—
CsSn_0.875_Cu_0.125_I_3_	12.04	5.90	15.22	2.04	0.28	—
CsSn_0.75_Cu_0.25_I_3_	14.03	4.82	13.62	2.90	0.27	—
CsSn_0.5_Cu_0.5_I_3_	17.57	8.52	22.02	2.02	0.30	—
CsCuI_3_	22.39	8.55	22.75	2.61	0.33	—

The evaluated bulk modulus, shear modulus, Young's modulus, Pugh's ratio, and Poisson's ratio of the pure and Cu-doped CsSnI_3_ samples are presented in Table 5. The bulk modulus values are identified that pure and Cu-doped CsSnI_3_ samples are flexible and soft. Therefore, these metal halide perovskites can easily be made into a suitable thin film for optoelectronic applications especially for solar cells. Bulk modulus to shear modulus ratio (*B*/*G*) is called Pugh's ratio and Poisson's ratio both can identify the ductility/brittleness nature of a material.^49,50^ The critical value of Pugh's and Poisons distinguish the brittle materials from ductile ones. If the Pugh's (0.26) and Poisson's ratio (1.75) values are higher than critical values, then the sample is said to be in ductile types, otherwise, it is brittle types. The ductile nature is tended to reduce with Cu-doping concentrations. Cauchy pressure predicted that pristine and Cu substitution doped CsSnI_3_ samples are ductile in nature. It can be seen from single and polycrystalline; elastic properties (Tables 4 and 5) are changed with Cu-doping concentrations. Notable, the single elastic properties of Cu-doped CsSnI_3_ samples are nearly similar with pristine CsSnI_3_ sample. The mechanical stability and ductility natures imply that the pure and Cu-doped CsSnI_3_ samples are perfect for the thin films industry.

### 3.4. Optical properties

To understand the optical behaviors, we simulated optical absorption (*α*), conductivity (σ), and dielectric constant (*ε*) for pristine and Cu-doped CsSnI_3_ samples. The complex dielectric function is given by the following equations.^51^

6 *ε*(*ω*) = *ε*_1_(*ω*) + *iε*_2_(*ω*) = *N*^2^

Here, *ε*_1_(*ω*), *iε*_2_(*ω*) and *N* represents the real and imaginary part of the dielectric constant and complex refractive index respectively.

The complex dielectric function is expressed by the following equations^52^7
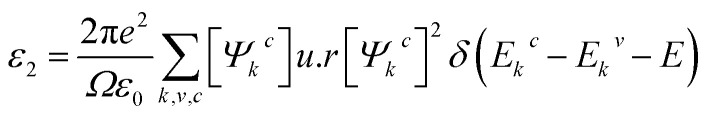
Where, the symbol *ω*, *e*, *Ω*, and *u* refer the phonon frequency, electronic charge, unit cell volume, and unit volume along the polarization of the incident electric field.

To see optical nature, we used photon energy, *E*, 0 to 20 eV. In this manuscript, the Gaussian smearing of 0.5 eV was used for all simulations. The optical properties calculation were taken in the {100} plane orientation. A scissor value is applied of 0.68 eV, which is the disparity between experimental and theoretical band gap energy.

**Fig. 7:**
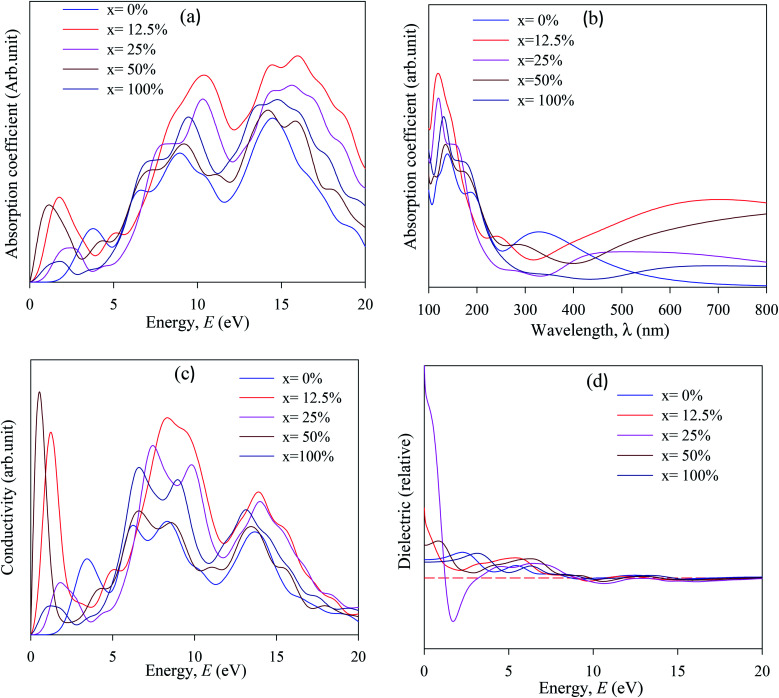
The simulated optical properties (a) absorbance *vs.* energy, (b) absorbance *vs.* wavelength, (c) optical conductivity, and (d) imaginary part of dielectric function of CsSn_1−*x*_Cu_*x*_I_3_, (*x* = 0, 0.125, 0.25, 0.5, 1).

The simulated absorption spectra of pristine and Cu-doped CsSnI_3_ are presented in Fig. 7(a and b). The optical absorption coefficient *α*(*ω*) gives the information about the amount of light entrance with a particular wavelength into solid materials.^28^

It also gives information about solar spectrum energy which is most important for devices application, especially for solar cells. First, absorption peak is more essential for device applications. The absorption spectra were taken in the range of 100–800 nm to investigate the optical behavior in the UV-vis. and visible wave length (*λ*) range. Accordingly, the absorption spectra are shifted to the lower energy region (redshift) compared to pure and Cu-doped CsSnI_3_. The absorption spectra confirmed that Cu has a large influence on the band gap energy and finally decreased the band gap. The absorption band is shifted towards the lower energy region due to Sn and Cu ions created defect energy of Cu 3d and Sn 4d orbitals.

Stronger optical absorption spectra identify increase photovoltaic efficiency. Hence the band gap energy transferred towards the visible region and the maximum absorption peak occurred in the UV region, which indicates that the pure and Cu-doped CsSnI_3_ samples are potential candidates for the optoelectronics industry. The absorption spectra threshold energy is significantly higher than the simulated band gap, which is indicating that the CsSnI_3_ sample has direct band gap nature and reverses at Cu-doped CsSnI_3_ samples. The first absorption peak stay at 1.0–4.5 eV energy regions, which demonstrated that pure and Cu-doped CsSnI_3_ samples are perfect for photoelectric device applications. The simulated optical conductivity (*σ*) are presented in Fig. 7(c). The optical conductivity is an essential parameter for identifying how the amount of electromagnetic wave response in a substance. Moreover, the optical conductivity (*σ*) refers to the number of photons that pass through the substance. The optical conductivity and absorption spectra have a similar structure, as presented in Fig. 7(a–c), due to the escape of electron and photon from the valence band to the conduction band when it absorbs energy. The optical conductivity of Cu-doped CsSnI_3_ occurs at lower energy compared with pristine CsSnI_3_. Optical conductivity at low energy of Cu-doped CsSnI_3_ perovskites makes them potential candidate materials for applications in optoelectronic especially solar cells devices. Optical conductivity results confirm that Cu-doped CsSnI_3_ samples have a low band gap compared with their pristine CsSnI_3_. The dielectric function gives information about the amount of electromagnetic radiation response in a solid substance.^53^ The imaginary part of the dielectric function (*ε*_2_) is similar to electron excitation. Notable, the first peak of the imaginary part of the dielectric function (*ε*_2_) occurs at <1.5 eV, at Cu-doped CsSnI_3_ samples, which indicates that the intra-band transition has occurred and *vice versa* at pure CsSnI_3_. The overall optical properties recommend that Cu-doped CsSnI_3_ is perfect for optoelectronic especially solar cell applications.

## 4. Conclusions

In this work, we have applied the density functional theory (DFT) simulations to calculate structural, electronic, mechanical, and optical properties of pure and Cu-doped CsSnI_3_ samples. The structural parameters lattice constants *a*, and cell volumes, *V* are well-matched with previously published work. The simulated band structure reveals that the pure CsSnI_3_ sample has a direct band gap nature semiconductor. In the case of Cu-doped CsSnI_3_ samples, the band gap energy transferred towards direct to indirect. The Pugh's and Poisson's ratio refers that the pure and Cu-doped CsSnI_3_ samples are to be fabricated easily in the thin films industry. The absorption edge is transferred to the lower energy regions with Cu-doping concentrations. The dielectric properties manifested that inter-band transferred towards the intra-band due to changes of the band gap energy at Cu-doped samples. A combined evaluation of the structural, electronic, mechanical, and optical properties recommend that eco-friendly CsSn_1−*x*_Cu_*x*_I_3_ (*x* = 0, 0.125, 0.25, 0.5, 1) perovskite is a suitable candidate materials for photovoltaic and optoelectronic device applications.

## Author contributions

M. N. Islam: conceptualization, data curation, investigation, methodology, software, formal analysis, writing-original draft, J. Podder: supervision, data curation, formal analysis, review and editing, M. L. Ali: formal analysis, software.

## Conflicts of interest

The authors declare no conflict of interest.

## Supplementary Material
